# Functional deficits after anterior cruciate ligament injury are associated with altered neuromuscular control: a cross-sectional study

**DOI:** 10.3389/fmed.2026.1945491

**Published:** 2026-09-16

**Authors:** Hang Yu, Shaochen Qu, Peiyao Liang, Shiyi Tong, Xing Xing, Lihang Zhang, Lin Guo

**Affiliations:** 1 State Key Laboratory of Trauma, Burn and Combined Injury, Department of Orthopedics/Sports Medicine Center, First Affiliated Hospital of Army Medical University, Chongqing, China; 2 Department of Traditional Chinese Medicine Rehabilitation, Jiangbei Campus, First Affiliated Hospital of Army Medical University, Chongqing, China; 3 School of Public Health, Peking University, Beijing, China

**Keywords:** anterior cruciate ligament, biomechanical phenomena, electromyography, muscle strength, neuromuscular control

## Abstract

**Objectives:**

Persistent muscle weakness after anterior cruciate ligament (ACL) injury may coexist with altered antagonist coactivation, but how these neuromuscular deficits differ according to injury chronicity and relate to patient-reported knee function remains incompletely characterized. This study aimed to compare knee muscle strength and antagonist coactivation among acute and chronic ACL injury groups, and healthy controls, and to evaluate the associations among muscle strength, antagonist coactivation, and Knee injury and Osteoarthritis Outcome Score (KOOS) subscale scores.

**Methods:**

This study included 114 patients with unilateral isolated ACL rupture [55 acute (<6 weeks) and 59 chronic (>6 months)] and 57 healthy controls. Maximal voluntary isometric contraction and concentric isokinetic knee extension and flexion at 60°/s were assessed. Surface electromyography quantified hamstring and quadriceps antagonist coactivation. KOOS subscales were collected in the ACL-injured groups. Group and sex comparisons, Spearman correlations, and multiple linear regression analyses were performed. Study design: Cross-sectional study; Level of Evidence: III.

**Results:**

Affected-limb extension peak torque was lowest in the acute group and remained lower in the chronic group than in controls for both male participants (2.23 ± 0.64, 3.05 ± 0.60, and 3.48 ± 0.59 N⋅m⋅kg^–1^, respectively) and female participants (1.51 ± 0.58, 2.21 ± 0.61, and 3.05 ± 0.56 N⋅m⋅kg^–1^; all *P* ≤ 0.001). Combined hamstring coactivation was greater in ACL-injured participants and highest in the chronic group (male: 14.32 ± 4.91%, 23.05 ± 5.87%, and 7.89 ± 4.34%; female: 27.18 ± 5.25%, 41.28 ± 6.34%, and 16.54 ± 5.28%; all *P* < 0.001). Extension average peak torque correlated positively with all KOOS subscales (*r* = 0.381–0.562), whereas combined hamstring coactivation correlated negatively with all KOOS subscales (*r* = −0.326 to −0.541; *P* < 0.05).

**Conclusion:**

ACL injury was associated with lower knee strength and greater antagonist coactivation, with distinct patterns according to injury chronicity. Strength deficits were most pronounced in patients with acute ACL injury, whereas antagonist coactivation was generally greatest in patients with chronic ACL injury. Greater hamstring coactivation and lower knee extension torque were associated with worse patient-reported knee function.

## 1 Introduction

Anterior cruciate ligament (ACL) rupture is a common sports-related injury (1). Persistent quadriceps weakness is frequently observed after ACL injury despite rehabilitation and is commonly attributed to arthrogenic muscle inhibition (AMI). AMI is driven by altered afferent signaling associated with pain, inflammation, joint effusion, and impaired mechanoreceptor function (2, 3).

ACL deficiency can increase shear forces across the knee and promote greater coactivation of antagonist muscles (4). During agonist contraction, this coactivation may serve as a protective strategy by increasing joint contact forces and mechanical impedance, thereby contributing to dynamic knee stability (5, 6). However, excessive or persistent coactivation may be metabolically inefficient and may increase articular loading, potentially accelerating joint degeneration (7).

The incidence of ACL injury is higher in female than in male athletes, a difference attributed to interacting anatomic, neuromuscular, biomechanical, and hormonal factors (8–10). Nevertheless, sex-related differences in strength and neuromuscular function after ACL injury remain insufficiently characterized. The optimal timing of surgical intervention also remains debated (11–13), and changes in strength and neuromuscular function over time may influence preoperative function, postoperative recovery, and rehabilitation planning (14). Characterizing differences between acute ( < 6 weeks after injury) (15) and chronic ( > 6 months after injury) ACL rupture may therefore inform individualized rehabilitation and surgical decision-making.

Muscle coactivation measured during standardized strength testing provides a reproducible approach to assessing neuromuscular control. Neuromuscular control reflects sensorimotor integration of somatosensory, visual, and vestibular inputs and the resulting motor responses (16). Quantifying antagonist coactivation during controlled strength tasks may therefore help identify interindividual differences in compensatory motor strategies and clarify whether these strategies are associated with muscle weakness and patient-reported knee function.

The purpose of this study was to compare knee muscle strength and antagonist activation patterns among patients with acute ACL injury, patients with chronic ACL injury, and healthy controls, and to evaluate the associations among muscle strength, antagonist coactivation, and Knee injury and Osteoarthritis Outcome Score (KOOS) subscale scores. We hypothesized that ACL-injured participants would demonstrate lower muscle strength and greater antagonist coactivation than healthy controls, that these neuromuscular patterns would differ between the acute and chronic injury groups, and that lower muscle strength and greater antagonist coactivation would be associated with worse KOOS subscale scores.

## 2 Materials and methods

### 2.1 Participants

This cross-sectional observational study prospectively recruited 114 patients aged 18–45 years with a unilateral, isolated, primary ACL rupture from the outpatient department of a university-affiliated hospital between September 2025 and January 2026 (Figure 1). The acute group comprised 55 patients assessed within 6 weeks of injury (31 male and 24 female), and the chronic group comprised 59 patients assessed more than 6 months after injury (33 male and 26 female). ACL rupture was confirmed by magnetic resonance imaging, and all patients had a Lachman test grade of II or III. The control group included 57 healthy participants aged 18–45 years (27 male and 30 female). Patients were excluded if they had (1) a time since ACL rupture between 6 weeks and 6 months; (2) a previous lower-extremity injury or surgery; (3) a concomitant meniscal or collateral ligament injury on magnetic resonance imaging or another orthopedic disorder affecting the hip, knee, or ankle; (4) knee swelling or effusion that restricted range of motion or prevented completion of strength testing; or (5) a neurologic disorder, including Parkinson disease, vertigo, or stroke. The study was approved by the Medical Ethics Committee of the First Affiliated Hospital of Army Medical University (approval No. (A)KY2025142) and was conducted in accordance with the Declaration of Helsinki. All participants provided written informed consent.

**FIGURE 1:**
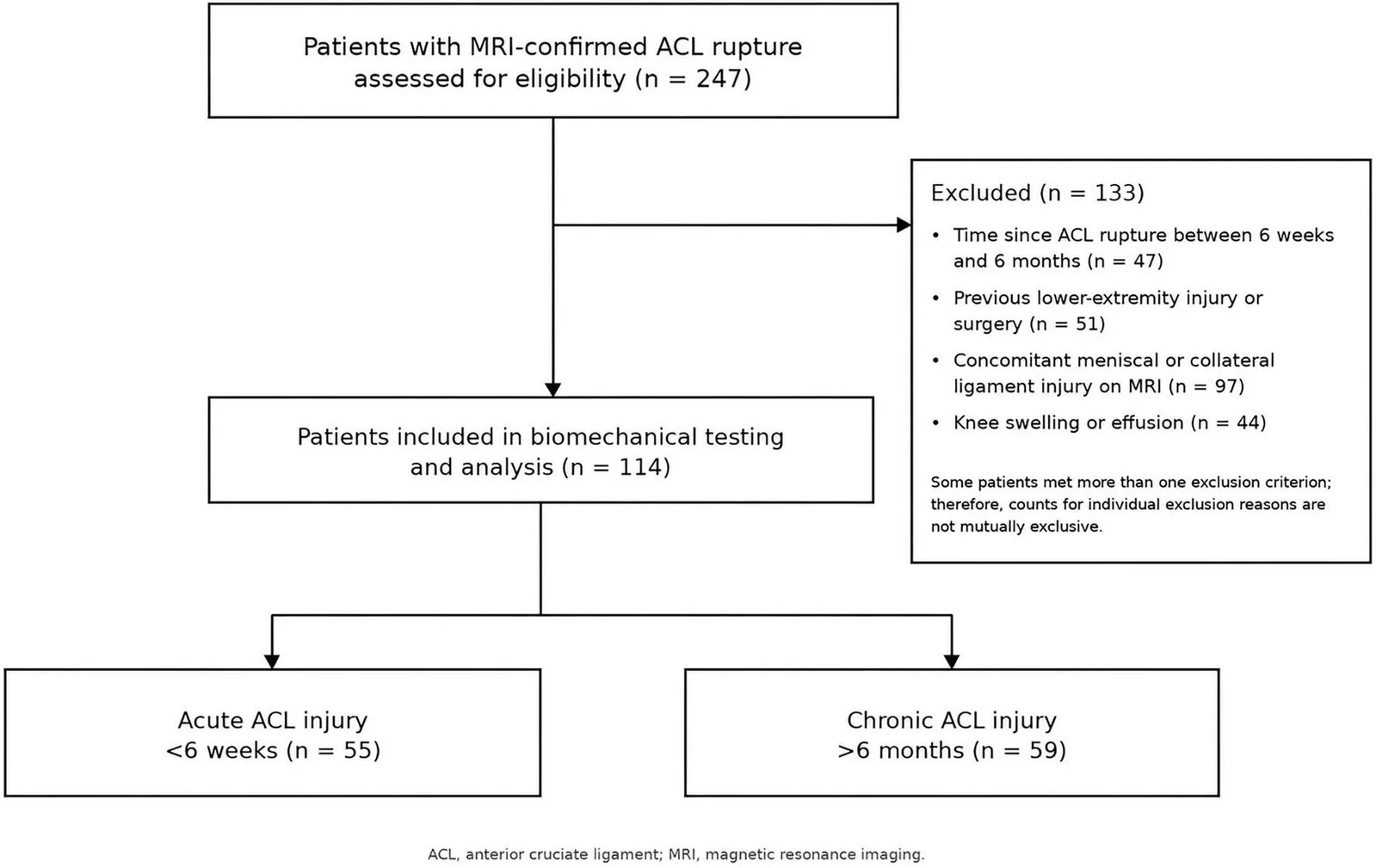
Flow diagram of ACL-injured patient screening and allocation.

Given the exploratory cross-sectional design and the lack of reliable prior effect size estimates for biomechanical parameters, no formal a priori sample size calculation was performed.

### 2.2 KOOS assessment

The KOOS is a 42-item, knee-specific patient-reported outcome measure comprising 5 subscales: Pain, Symptoms, Activities of Daily Living (ADL), Sport and Recreation (Sport/Rec), and knee-related Quality of Life (QoL). Each subscale is transformed to a 0–100 score, with 0 indicating extreme knee problems and 100 indicating no knee problems (17).

### 2.3 Muscle strength assessment

Knee strength was assessed using an isokinetic dynamometer (IsoMed 2000; D&R Ferstl GmbH, Germany), which has demonstrated excellent reliability for measuring knee extension peak torque (18). Participants were seated with the trunk and hips flexed to 90° and the thigh positioned parallel to the floor. The dynamometer axis was aligned with the lateral femoral epicondyle, and the resistance pad was secured to the distal lower leg. Participants completed 7 maximal concentric knee extension and flexion repetitions at an angular velocity of 60°/s. They were then repositioned with the knee at 60° of flexion for maximal voluntary isometric contraction (MVIC) testing. Standardized verbal encouragement and real-time visual feedback from the torque-time curve were provided throughout testing (19). For control participants, the non-dominant limb was used as the comparator for the affected limb and the dominant limb as the comparator for the contralateral limb.

Voltage signals were converted to torque (N⋅m) using a site-specific calibration factor (18) and normalized to body mass (N⋅m⋅kg^–1^) to facilitate between-participant comparisons. The biomechanical outcomes were peak torque (PT), defined as the maximum value on the torque-time curve; average peak torque (APT), defined as the mean PT across the 7 repetitions; rate of torque development during the first 100 ms (RTD100); and average power (AP), defined as total work divided by movement time. The limb symmetry index (LSI) and hamstring-to-quadriceps ratio (H:Q) were calculated using Equations 1, 2, respectively.


$$L\,S\,I=\frac{A\,C\,L\,a\,f\,f\,e\,c\,t\,e\,d/N\,o\,n-d\,o\,m\,i\,n\,a\,n\,t\,s\,i\,d\,e}{C\,o\,n\,t\,r\,a\,l\,a\,t\,e\,r\,a\,l/D\,o\,m\,i\,n\,a\,n\,t\,s\,i\,d\,e}\times 100\%$$ (1)

$$H:Q=\frac{h\,a\,m\,s\,t\,r\,i\,n\,g\,s\,t\,r\,e\,n\,g\,t\,h\,\left(f\,l\,e\,x\,o\,r\right)}{q\,u\,a\,d\,r\,i\,c\,e\,p\,s\,s\,t\,r\,e\,n\,g\,t\,h\,\left(e\,x\,t\,e\,n\,s\,o\,r\right)}\times 100\%$$ (2)

### 2.4 Surface electromyography

During isokinetic testing, activation of the medial hamstrings (MH), lateral hamstrings (LH), vastus medialis (VM), and vastus lateralis (VL) was recorded using a 16-channel surface electromyography (sEMG) system (Noraxon Ultium; Noraxon, United States). The skin was shaved and cleaned with alcohol to reduce impedance. Electrodes were placed parallel to the underlying muscle fibers with an interelectrode distance of 2 cm. Signals were preamplified at a gain of 10, band-pass filtered at 20–450 Hz, and sampled at 2,000 Hz using a 24-bit acquisition card. After full-wave rectification, the signals were smoothed using a root mean square algorithm with a 200-ms moving window.

The sEMG amplitude of each antagonist muscle was normalized to its maximum activation when functioning as the agonist, allowing antagonist activation to be expressed relative to the maximum task-specific agonist activation of the same muscle during the isokinetic trials (16). Specifically, in the same isokinetic testing trials, hamstring activation during knee extension was normalized to the maximum hamstring activation obtained during knee flexion, whereas quadriceps activation during knee flexion was normalized to the maximum quadriceps activation obtained during knee extension. This task-specific, within-muscle normalization approach was used to reduce interindividual variability related to electrode placement and raw signal amplitude. Mean antagonist activation across the 7 repetitions was used as the coactivation measure (20). Antagonist amplitude was defined as the activation of the medial or lateral antagonist during maximal quadriceps or hamstring torque production. Baseline amplitude was defined as the mean amplitude during a predefined resting baseline period. Baseline noise was corrected using the root-sum-of-squares method shown in Equation 3. Combined hamstring (CH) and combined quadriceps (CQ) coactivation were calculated as the root mean square of the medial and lateral muscle activation values, as shown in Equation 4 (20).


A⁢n⁢t⁢a⁢g⁢o⁢n⁢i⁢s⁢t⁢C⁢o⁢a⁢c⁢t⁢i⁢v⁢a⁢t⁢i⁢o⁢n= (3)

$$\sqrt{{\left(A\,n\,t\,a\,g\,o\,n\,i\,s\,t\,A\,m\,p\,l\,i\,t\,u\,d\,e\right)}^{2}-{\left(B\,a\,s\,e\,l\,i\,n\,e\,A\,m\,p\,l\,i\,t\,u\,d\,e\right)}^{2}}$$

C⁢o⁢m⁢b⁢i⁢n⁢e⁢d⁢C⁢o⁢a⁢c⁢t⁢i⁢v⁢a⁢t⁢i⁢o⁢n= (4)

$$\sqrt{\frac{{\left(M\,e\,d\,i\,a\,l\,C\,o\,a\,c\,t\,i\,v\,a\,t\,i\,o\,n\right)}^{2}+{\left(L\,a\,t\,e\,r\,a\,l\,C\,o\,a\,c\,t\,i\,v\,a\,t\,i\,o\,n\right)}^{2}}{2}}$$

Two coactivation ratios were also calculated. The lateral-to-medial antagonist activation ratio was calculated for the hamstrings during knee extension (LH/MH) and for the quadriceps during knee flexion (VL/VM). The combined hamstring-to-quadriceps coactivation ratio was calculated as CH/CQ.

### 2.5 Statistical analysis

Statistical analyses were performed using SPSS Statistics, version 26.0 (IBM Corp). All tests were two-sided, and statistical significance was set at *P* < 0.05. Normality and homogeneity of variance were assessed using the Shapiro-Wilk and Levene tests, respectively. Between-sex and between-injury-stage comparisons were performed using independent-samples *t*-tests or Wilcoxon rank-sum tests, as appropriate. Sex-stratified comparisons were conducted as secondary exploratory analyses. Differences among the acute, chronic, and control groups were assessed using 1-way analysis of variance or the Kruskal-Wallis H test. When an overall group difference was identified, Tukey or Bonferroni-adjusted *post hoc* comparisons were performed, as appropriate. Spearman rank correlation was used to evaluate associations among muscle strength, coactivation measures, and KOOS subscale scores. Multiple linear regression analyses were then performed to identify biomechanical variables associated with each KOOS subscale. Candidate predictors were restricted to variables that were significant in the Spearman analysis. The same approach was used to examine associations between isokinetic strength outcomes and coactivation measures. All regression models were adjusted for sex, height, and body mass to account for potential anthropometric confounding.

## 3 Results

Participant characteristics are summarized in Table 1. Age and body mass index did not differ significantly among the acute, chronic, and control groups or between male and female participants. Male participants were taller and heavier than female participants (both *P* < 0.001), whereas height and body mass did not differ significantly among groups. Time since injury was longer in the chronic group than in the acute group (*P* < 0.001) and did not differ by sex. Across all 5 KOOS subscales, the chronic group reported higher scores than the acute group (*P* = <0.001–0.043), and female participants reported lower scores than male participants (*P* = <0.001–0.027).

**TABLE 1:** Demographic characteristics of the participants.

Characteristics	Acute group (*n* = 55)		Chronic group (*n* = 59)		Control group (*n* = 57)		*P*-value	
	Male (*n* = 31)	Female (*n* = 24)	Male (*n* = 33)	Female (*n* = 26)	Male (*n* = 27)	Female (*n* = 30)	P1	P2
Age (years)	28.3 ± 5.9	30.5 ± 7.4	32.4 ± 8.2	29.3 ± 7.6	31.5 ± 8.9	32.1 ± 7.2	0.055	0.450
Height (cm)	175.4 ± 5.2	162.3 ± 5.1*	173.8 ± 6.2	161.5 ± 7.3*	172.8 ± 6.9	160.4 ± 5.7*	0.377	0.630
Body mass (kg)	71.5 ± 8.7	59.2 ± 7.3*	75.5 ± 9.4	62.1 ± 8.4*	74.5 ± 8.5	60.2 ± 7.8*	0.481	0.303
BMI (kg⋅m^–2^)	22.8 ± 4.0	23.5 ± 3.5	23.1 ± 4.4	24.0 ± 3.6	22.5 ± 3.9	23.4 ± 3.8	0.705	0.417
Time since injury (months)	0.9 ± 0.4	0.7 ± 0.3	26.3 ± 10.0	29.3 ± 12.0	/	/	< 0.001^a^	<0.001^a^
KOOS-pain	56.2 ± 19.2	44.3 ± 19.3*	79.3 ± 25.7	63.4 ± 26.7*	/	/	< 0.001^a^	0.012^a^
KOOS-symptoms	49.1 ± 20.3	37.5 ± 21.4*	74.1 ± 23.1	52.4 ± 24.2*	/	/	< 0.001^a^	0.007^a^
KOOS-ADL	40.3 ± 15.2	30.2 ± 14.2*	57.3 ± 25.1	42.2 ± 23.8*	/	/	< 0.001^a^	0.043^a^
KOOS-sports/Rec	47.2 ± 24.3	31.1 ± 25.3*	70.7 ± 23.3	56.4 ± 23.4*	/	/	< 0.001^a^	<0.001^a^
KOOS-QoL	50.7 ± 15.3	35.3 ± 14.1*	73.4 ± 20.1	61.2 ± 23.1*	/	/	< 0.001^a^	<0.001^a^

Data are presented as mean ± SD. Group comparisons for male and female participants are indicated by P1 and P2, respectively. ADL, Activities of Daily Living; BMI, body mass index; KOOS, Knee injury and Osteoarthritis Outcome Score; QoL, Quality of Life; Sport/Rec, Sport and Recreation.

^a^Significant difference between the acute and chronic groups (*P* < 0.05).

*Significant difference compared with male participants (*P* < 0.05).

### 3.1 MVIC test

During knee extension, affected-limb PT, RTD100, LSI_*PT*_, and LSI_*RTD100*_ were lower in the ACL-injured groups than in controls (*P* = <0.001–0.008). These outcomes were also lower in the acute group than in the chronic group (all *P* < 0.001) and were generally lower in female than in male participants (*P* = <0.001–0.011). In contrast, contralateral-limb extension PT and RTD100 were higher in the acute group than in the chronic and control groups (*P* = <0.001–0.043), with no significant difference between the chronic and control groups. During knee flexion, affected-limb PT, RTD100, LSI_*PT*_, and LSI_*RTD100*_ were lower in the acute group than in both the chronic and control groups (all *P* < 0.001). Compared with controls, the chronic group had lower affected-limb RTD100 and LSIRTD100 (both *P* < 0.001), whereas PT and LSI_*PT*_ did not differ significantly. No significant sex-related differences were observed for affected-limb flexion PT, RTD100, LSI_*PT*_, or LSI_*RTD100*_. Contralateral-limb flexion outcomes were higher in the acute group than in the chronic and control groups (*P* = <0.001–0.049), with no significant difference between the chronic and control groups (Table 2 and Supplementary Table 1).

**TABLE 2:** Maximal voluntary isometric contraction strength outcomes.

Outcome	Acute group (*n* = 55)		Chronic group (*n* = 59)		Control group (*n* = 57)		*P*-value	
	Affected side	Contralateral side	Affected side	Contralateral side	Non-dominant side	Dominant side	P1	P2
**Extension**								
PT (N⋅m⋅kg–1)								
Male	2.23 ± 0.64	4.10 ± 0.75	3.05 ± 0.60	3.52 ± 0.63	3.48 ± 0.59	3.65 ± 0.63	< 0.001^abc^	<0.001^ab^
Female	1.51 ± 0.58*	3.62 ± 0.70	2.21 ± 0.61*	2.97 ± 0.69	3.05 ± 0.56*	3.11 ± 0.55	0.001^abc^	0.003^ab^
RTD100 (N⋅m⋅s–1⋅kg–1)								
Male	8.34 ± 3.65	21.32 ± 4.95	14.78 ± 3.01	17.93 ± 4.02	17.67 ± 3.43	18.11 ± 4.51	< 0.001^abc^	<0.001^ab^
Female	5.45 ± 2.88*	19.45 ± 4.12	8.91 ± 3.15*	14.24 ± 4.57	15.02 ± 2.89*	15.73 ± 4.11	< 0.001^abc^	<0.001^ab^
**Flexion**								
PT (N⋅m⋅kg–1)								
Male	1.13 ± 0.49	2.33 ± 0.61	1.80 ± 0.42	2.02 ± 0.45	1.88 ± 0.35	1.99 ± 0.43	< 0.001^ab^	0.039^ab^
Female	1.05 ± 0.46	2.16 ± 0.55	1.63 ± 0.39	1.85 ± 0.42	1.77 ± 0.33	1.83 ± 0.31	< 0.001^ab^	0.014^ab^
RTD100 (N⋅m⋅s–1⋅kg–1)								
Male	3.34 ± 1.45	9.48 ± 1.33	5.74 ± 1.28	8.45 ± 1.13	8.21 ± 1.09	8.50 ± 1.29	< 0.001^abc^	<0.001^ab^
Female	2.71 ± 1.37	8.81 ± 1.35	5.11 ± 1.29	7.56 ± 1.25	7.63 ± 1.35	7.65 ± 1.06	< 0.001^abc^	0.008^ab^

Data are presented as mean ± SD. Group comparisons for the affected/non-dominant and contralateral/dominant limbs are indicated by P1 and P2, respectively. PT, peak torque; RTD100, rate of torque development from 0 to 100 ms.

^a^Significant difference between the acute and chronic groups (*P* < 0.05).

^b^Significant difference between the acute and control groups (*P* < 0.05).

^c^Significant difference between the chronic and control groups (*P* < 0.05).

*Significant difference compared with male participants (*P* < 0.05).

### 3.2 Isokinetic strength test

During knee extension, affected-limb APT, AP, LSI_*APT*_, and LSI_*AP*_ were lower in both ACL-injured groups than in controls (*P* = <0.001–0.007) and lower in the acute group than in the chronic group (all *P* < 0.001). These extension outcomes were also lower in female than in male participants (*P* = <0.001–0.004). During knee flexion, affected-limb APT and LSI_*APT*_ were lower in both ACL-injured groups than in controls and lower in the acute group than in the chronic group (all *P* < 0.001). Affected-limb AP and LSI_*AP*_ were lower in ACL-injured participants than in controls (all *P* < 0.001), but did not differ significantly between the acute and chronic groups. Female participants had lower affected-limb flexion outcomes than male participants (*P* = <0.001–0.013). The affected-limb H:Q ratio was higher in the ACL-injured groups than in controls (*P* = <0.001–0.007), higher in the acute group than in the chronic group (*P* = 0.002 and 0.012 for male and female participants, respectively), and higher in male than in female participants (*P* < 0.001). No significant group differences were observed for contralateral-limb isokinetic strength outcomes (Table 3 and Supplementary Table 1).

**TABLE 3:** Isokinetic knee strength outcomes at 60°/s.

Outcome	Acute group (*n* = 55)		Chronic group (*n* = 59)		Control group (*n* = 57)		*P*-value	
	Affected side	Contralateral side	Affected side	Contralateral side	Non-dominant side	Dominant side	P1	P2
**Extension**								
APT (N⋅m⋅kg–1)								
Male	1.35 ± 0.51	2.51 ± 0.36	1.87 ± 0.45	2.52 ± 0.38	2.53 ± 0.40	2.56 ± 0.46	< 0.001^abc^	0.193
Female	0.84 ± 0.45*	2.22 ± 0.40	1.34 ± 0.43*	2.15 ± 0.41	2.09 ± 0.42*	2.21 ± 0.38	< 0.001^abc^	0.803
AP (W⋅kg–1)								
Male	0.96 ± 0.16	2.01 ± 0.14	1.28 ± 0.11	1.93 ± 0.15	1.96 ± 0.14	1.98 ± 0.13	< 0.001^abc^	0.662
Female	0.82 ± 0.14*	1.84 ± 0.10	1.05 ± 0.16*	1.85 ± 0.20	1.79 ± 0.18*	1.82 ± 0.15	< 0.001^abc^	0.694
**Flexion**								
APT (N⋅m⋅kg–1)								
Male	1.07 ± 0.28	1.62 ± 0.31	1.27 ± 0.24	1.55 ± 0.31	1.56 ± 0.24	1.60 ± 0.32	< 0.001^abc^	0.321
Female	0.55 ± 0.19*	1.14 ± 0.27	0.79 ± 0.22*	1.11 ± 0.23	1.05 ± 0.20*	1.13 ± 0.22	< 0.001^abc^	0.348
AP (W⋅kg–1)								
Male	0.68 ± 0.24	1.15 ± 0.25	0.73 ± 0.27	1.16 ± 0.23	1.13 ± 0.26	1.18 ± 0.23	< 0.001^bc^	0.517
Female	0.48 ± 0.21*	0.87 ± 0.27	0.51 ± 0.26*	0.86 ± 0.21	0.82 ± 0.26*	0.87 ± 0.25	< 0.001^bc^	0.379
H:Q (%)								
Male	79.13 ± 11.34	63.45 ± 10.23	68.27 ± 9.59	61.25 ± 9.87	60.84 ± 8.73	59.27 ± 9.13	< 0.001^abc^	0.086
Female	68.35 ± 10.91*	49.59 ± 11.15	59.25 ± 9.58*	49.48 ± 8.91	49.12 ± 9.87*	50.99 ± 10.15	< 0.001^abc^	0.292

Data are presented as mean ± SD. Group comparisons for the affected/non-dominant and contralateral/dominant limbs are indicated by P1 and P2, respectively. AP, average power; APT, average peak torque; H:Q, hamstring-to-quadriceps ratio.

^a^Significant difference between the acute and chronic groups (*P* < 0.05).

^b^Significant difference between the acute and control groups (*P* < 0.05).

^c^Significant difference between the chronic and control groups (*P* < 0.05).

*Significant difference compared with male participants (*P* < 0.05).

### 3.3 sEMG during isokinetic testing

Antagonist coactivation in the affected limb was greater in ACL-injured participants than in controls (*P* = <0.001–0.034) and was generally greater in female than in male participants (*P* = <0.001–0.012). Compared with the acute group, the chronic group demonstrated greater CH, LH, MH, VL, LH/MH, VL/VM, and CH/CQ values in the affected limb (*P* = <0.001–0.015); CQ and VM did not differ significantly between the acute and chronic groups. Male participants had higher affected-limb LH/MH and VL/VM ratios than female participants (*P* = <0.001 and 0.004). No corresponding group differences were observed in the contralateral limb (Table 4 and Supplementary Table 2).

**TABLE 4:** Antagonist muscle coactivation outcomes.

Outcome	Acute group (*n* = 55)		Chronic group (*n* = 59)		Control group (*n* = 57)		*P*-value	
	Affected side	Contralateral side	Affected side	Contralateral side	Non-dominant side	Dominant side	P1	P2
**Extension**								
CH (%)								
Male	14.32 ± 4.91	8.74 ± 3.59	23.05 ± 5.87	9.39 ± 4.01	7.89 ± 4.34	7.54 ± 4.91	< 0.001^abc^	0.918
Female	27.18 ± 5.25*	15.03 ± 4.28	41.28 ± 6.34*	16.03 ± 6.48	16.54 ± 5.28*	17.21 ± 5.59	< 0.001^abc^	0.164
LH (%)								
Male	17.73 ± 6.23	9.45 ± 5.15	32.71 ± 5.02	11.73 ± 5.49	10.23 ± 4.68	9.73 ± 5.29	< 0.001^abc^	0.482
Female	37.51 ± 7.15*	24.87 ± 4.49	57.31 ± 6.28*	25.49 ± 4.41	25.78 ± 4.89*	23.41 ± 5.98	< 0.001^abc^	0.229
LH/MH								
Male	2.19 ± 0.21	1.85 ± 0.28	2.58 ± 0.24	1.93 ± 0.24	1.81 ± 0.29	1.87 ± 0.23	< 0.001^abc^	0.073
Female	1.94 ± 0.35*	1.57 ± 0.30	2.35 ± 0.37*	1.62 ± 0.28	1.55 ± 0.35*	1.51 ± 0.37	< 0.001^abc^	0.695
**Flexion**								
CQ (%)								
Male	10.08 ± 4.09	6.04 ± 3.78	9.87 ± 4.19	5.39 ± 4.23	6.03 ± 3.80	5.43 ± 4.01	< 0.001^bc^	0.316
Female	14.47 ± 3.78*	8.64 ± 3.34	14.09 ± 3.88*	8.48 ± 3.78	9.13 ± 3.21*	8.44 ± 3.53	< 0.001^bc^	0.701
VL (%)								
Male	13.54 ± 3.58	6.59 ± 3.37	16.47 ± 3.34	7.23 ± 2.89	7.24 ± 3.02	7.01 ± 3.18	< 0.001^abc^	0.392
Female	16.59 ± 5.91*	8.01 ± 4.11	21.48 ± 5.97*	8.41 ± 4.01	9.27 ± 4.02*	8.73 ± 4.13	< 0.001^abc^	0.955
VL/VM								
Male	1.75 ± 0.25	1.57 ± 0.29	1.99 ± 0.28	1.58 ± 0.31	1.52 ± 0.27	1.55 ± 0.23	< 0.001^abc^	0.869
Female	1.61 ± 0.23*	1.27 ± 0.22	1.83 ± 0.29*	1.23 ± 0.25	1.29 ± 0.20*	1.21 ± 0.18	< 0.001^abc^	0.442
CH/CQ								
Male	1.39 ± 0.21	1.21 ± 0.23	2.56 ± 0.24	1.28 ± 0.25	1.20 ± 0.28	1.18 ± 0.32	< 0.001^abc^	0.472
Female	1.96 ± 0.45*	1.60 ± 0.41	3.15 ± 0.54*	1.69 ± 0.43	1.67 ± 0.39*	1.69 ± 0.33	< 0.001^abc^	0.584

Data are presented as mean ± SD. Group comparisons for the affected/non-dominant and contralateral/dominant limbs are indicated by P1 and P2, respectively. CH, combined hamstrings; CH/CQ, combined hamstring-to-quadriceps coactivation ratio; CQ, combined quadriceps; LH, lateral hamstrings; LH/MH, lateral-to-medial hamstring coactivation ratio; VL, vastus lateralis; VL/VM, vastus lateralis-to-vastus medialis coactivation ratio.

^a^Significant difference between the acute and chronic groups (*P* < 0.05).

^b^Significant difference between the acute and control groups (*P* < 0.05).

^c^ Significant difference between the chronic and control groups (*P* < 0.05).

* Significant difference compared with male participants (*P* < 0.05).

### 3.4 Correlation analysis

Affected-limb extension PT, extension RTD100, flexion PT, flexion RTD100, and extension APT were positively correlated with KOOS Pain (*r* = 0.329–0.589; *P* < 0.05). CH and LH were negatively correlated with Pain (*r* = −0.326 and-0.591, respectively; *P* < 0.05). Extension APT and H:Q were positively correlated with KOOS Symptoms, ADL, and Sport/Rec (*r* = 0.346–0.562; *P* < 0.05). Flexion APT correlated positively with ADL (*r* = 0.327; *P* < 0.05), and extension AP correlated positively with Sport/Rec (*r* = 0.503; *P* < 0.05). Extension APT also correlated positively with QoL (*r* = 0.459; *P* < 0.05), whereas CH, LH, CQ, and CH/CQ correlated negatively with QoL (*r* = −0.408 to −0.541; *P* < 0.05) (Supplementary Table 3). Extension strength outcomes were predominantly negatively correlated with CH, LH, MH, LH/MH, and VL (*r* = −0.505 to −0.308; *P* < 0.05), whereas flexion strength outcomes were negatively correlated with CQ, VL, VM, and VL/VM (*r* = −0.512 to −0.318; *P* < 0.05). CH correlated positively with H:Q, CH/CQ correlated positively with flexion APT, flexion AP, and H:Q (*r* = 0.334–0.492; *P* < 0.05). CQ and VL/VM correlated negatively with H:Q (*r* = −0.465 and −0.435, respectively; *P* < 0.05) (Supplementary Table 4).

### 3.5 Multiple linear regression analysis

The regression models for the KOOS subscales are presented in Table 5. Each model included 2 or 3 biomechanical variables that were independently associated with the relevant KOOS subscale (*P*-values ranged from 0.003 to 0.036). Separate regression models were constructed for 5 isokinetic strength outcomes using coactivation measures that were significant in the Spearman analysis. Each model included 1–3 sEMG variables that were independently associated with the corresponding isokinetic strength outcome (*P*-values ranged from 0.002 to 0.037) (Table 6).

**TABLE 5:** Associations of muscle strength and sEMG parameters with KOOS subscale scores.

Predictor	B	β	*P*-value	95% CI
KOOS-Pain regression model				
PT_*E*_	21.674	0.711	0.009	15.34, 27.79
RTD100_*E*_	2.847	0.532	0.036	1.92, 3.95
APT_*E*_	18.495	0.512	0.004	14.96, 21.57
KOOS-symptoms regression model				
APT_*E*_	23.480	0.583	0.033	19.35, 29.54
H:Q	1.341	1.285	0.035	0.05, 2.97
KOOS-ADL regression model				
APT_*E*_	5.868	0.218	0.029	3.45, 7.56
H:Q	0.691	0.466	0.017	0.13, 1.25
KOOS-sport/recreation regression model				
APT_*E*_	33.358	0.616	0.032	16.35, 41.29
H:Q	1.547	0.563	0.023	0.57, 2.59
KOOS-QoL regression model				
CH	−2.542	−0.814	0.003	−3.19, −2.11
CH/CQ	−18.029	−0.445	0.012	−24.58, −12.78

B, unstandardized regression coefficient; β, standardized regression coefficient; CI, confidence interval; E, extension; F, flexion; KOOS, Knee injury and Osteoarthritis Outcome Score.

**TABLE 6:** Associations between isokinetic strength and sEMG parameters.

Predictor	B	β	*P*-value	95% CI
APTE regression model				
CH	−0.098	−0.943	0.009	−0.17, −0.01
LH	−0.109	−1.522	0.005	−0.19, −0.03
LH/MH	−1.130	−0.643	0.034	−3.98, −0.11
APE regression model				
CH	−0.044	−1.357	0.008	−0.12, −0.01
LH	−0.068	−3.026	0.011	−0.16, −0.03
APTF regression model				
CQ	−0.141	−1.842	0.026	−0.27, −0.06
VL/VM	−1.186	−1.011	0.020	−3.14, −0.59
APF regression model				
VL	−0.056	−0.730	0.010	−0.13, −0.01
H:Q regression model				
CH	1.979	0.864	0.002	1.02, 2.97
CQ	−1.834	−0.649	0.037	−3.79, −0.09
CH/CQ	9.783	0.252	0.009	6.02, 10.98

B, unstandardized regression coefficient; β, standardized regression coefficient; CI, confidence interval; E, extension; F, flexion.

## 4 Discussion

The principal findings were that ACL-injured participants had lower affected-limb muscle strength and greater antagonist coactivation than healthy controls, with different patterns according to injury chronicity. Strength deficits were most pronounced in the acute group, whereas antagonist coactivation was generally greatest in the chronic group. Female participants demonstrated lower affected-limb strength and greater coactivation than male participants. Lower strength and greater hamstring coactivation were also associated with worse KOOS scores. Together, these findings suggest that muscle weakness and altered neuromuscular control represent related but distinct features of functional impairment after ACL injury.

KOOS scores were lower in the acute group than in the chronic group and were lower in female than in male participants, consistent with previous studies (21–23). Because male and female participants differed in height and body mass, interpretation of sex-related differences should account for anthropometric and baseline strength characteristics. The acute ( < 6 weeks) and chronic ( > 6 months) groups were defined according to time since ACL injury. Previous studies suggest that pain, inflammation, and AMI are commonly observed shortly after ACL injury, whereas different neuromuscular control patterns have been reported in individuals with longer injury durations (3, 24). Because the present study was cross-sectional rather than longitudinal, the observed differences between groups should not be interpreted as within-patient changes over time.

Affected-limb quadriceps strength differed among the acute, chronic, and control groups during MVIC, whereas flexion PT did not differ significantly between the chronic and control groups (Table 2). This pattern supports previous evidence that quadriceps weakness is a predominant impairment after ACL rupture (2, 23, 25, 26). The lower affected-limb PT and KOOS scores in the acute group relative to the chronic group are consistent with the findings of Pei-An et al. (23). AMI is common after acute ACL injury, and psychological responses such as avoidance, intrusive thoughts, and hypervigilance may also affect maximal effort and function (27, 28). The lower strength observed in the acute group may be consistent with the presence of pain-related and inflammatory neuromodulation, joint effusion, and fear of reinjury reported in previous studies. The lower strength observed in the chronic group may be associated with differences in muscle morphology and motor control that have been reported in patients with longer injury durations (29, 30). These cross-sectional findings highlight injury chronicity as an important variable but require confirmation in prospective longitudinal studies.

The acute group also demonstrated greater contralateral-limb PT and RTD100 during MVIC than the chronic and control groups, together with lower LSI values. This pattern may indicate task-specific compensation through increased force output of the uninjured limb during maximal isometric testing. The absence of a similar pattern in the chronic group may indicate differences in bilateral motor control between injury stages, although this interpretation remains speculative (24, 31).

The affected-limb H:Q ratio was higher in the acute group than in the chronic group. Pei-An et al. (23) did not identify a significant difference in H:Q between acute and chronic ACL injury, possibly because the definition of acute injury differed between studies. In addition, contralateral-limb group differences were observed during MVIC but not during isokinetic testing, suggesting that the observed between-group patterns may be task dependent. Isokinetic testing requires continuous coordination of agonist and antagonist activation to maintain a constant angular velocity (16, 32), whereas MVIC emphasizes maximal instantaneous force and rapid torque development. The higher contralateral PT and RTD100 observed during MVIC but not during isokinetic testing suggest that the neuromuscular patterns identified after ACL injury may be task specific. Combining isometric and isokinetic assessments may provide a more complete characterization of these neuromuscular patterns.

During knee extension, ACL-injured participants demonstrated greater CH, LH, and LH/MH values than controls, indicating increased hamstring coactivation, with greater lateral than medial hamstring coactivation. Animal and human studies suggest that impaired proprioceptive input after ACL injury may alter central sensorimotor strategies and increase biceps femoris activation during demanding tasks (33, 34). Increased hamstring and quadriceps coactivation can reduce rotational, translational, and shear displacement of the knee (35). Thus, greater lateral hamstring coactivation may be consistent with a compensatory strategy related to dynamic knee stability (4, 6, 33, 35). However, increased hamstring coactivation has also been associated with subsequent cartilage deterioration (36), indicating that a strategy that improves short-term stability could increase long-term joint loading.

Quadriceps coactivation during knee flexion was also altered. VL and VL/VM were greater in ACL-injured participants than in controls, suggesting a shift toward lateral quadriceps activation. This finding is consistent with the work of Reed-Jones and Vallis (37) and may reflect compensation for rotational instability. Persistent imbalance between VL and VM activation could alter patellar tracking and contribute to patellofemoral symptoms (38). These findings suggest that the coordination and distribution of muscle activation around the knee may warrant consideration alongside muscle strength in future rehabilitation studies.

Female participants generally demonstrated lower affected-limb strength and greater coactivation than male participants. These differences should be interpreted cautiously because they may be associated with biological sex-related characteristics as well as unmeasured differences in preinjury activity level, sport type and exposure, hormonal status, and baseline muscle strength. The sex-related differences in muscle strength and coactivation levels revealed in this study provide a rationale for individualized assessment and rehabilitation (39, 40), but they should not yet be considered treatment targets without confirmation in interventional studies.

Extension APT and CH were consistently associated with KOOS subscale scores (Table 5), in agreement with previous evidence linking neuromechanical impairments to patient-reported function (41). Lower extension APT reflects reduced quadriceps force-generating capacity and was associated with worse Symptoms, ADL, Sport/Rec, and QoL scores. In contrast, greater CH reflects increased antagonist coactivation and was associated with greater pain and worse symptoms, function, and quality of life. Regression models relating isokinetic strength to sEMG outcomes further indicated that lower APT and AP were associated with greater antagonist coactivation during knee extension and flexion (Table 6). These findings are consistent with those of Pamukoff et al. (42), who reported that excessive antagonist coactivation may reduce net agonist force and joint torque. Because the data were cross-sectional, however, the direction of these associations cannot be established.

This study has several strengths. First, the inclusion of patients with acute ACL injury, patients with chronic ACL injury, and healthy controls enabled the characterization of neuromuscular profiles across groups defined by time since injury. Second, the combined use of maximal voluntary isometric contraction testing, isokinetic dynamometry, and surface electromyography provided complementary assessments of muscle force-generating capacity and antagonist coactivation. Third, bilateral testing allowed affected-limb deficits and potential contralateral neuromuscular patterns to be examined simultaneously. Finally, the integration of objective neuromuscular measurements with KOOS subscale scores provided clinically relevant information regarding the relationships between biomechanical impairments and patient-reported knee function.

This study has several limitations. The modest and unequal subgroup sizes, particularly after stratification by sex, may have limited statistical power and the precision of the estimated between-group differences. Although age did not differ significantly among the groups, residual confounding related to age and other baseline characteristics cannot be completely excluded. Its cross-sectional design compared separate acute and chronic cohorts and therefore cannot establish temporal changes or causal relationships. Testing was performed in a controlled laboratory environment; the dynamometer, visual display, and electrode leads may have altered movement behavior and limited ecological validity. Wearable sensors could help validate these findings during free-living and sport-specific tasks. EMG amplitude primarily reflects neural drive rather than direct mechanical force. The conversion of neural excitation to force is influenced by tendon stiffness, muscle fiber composition, fatigue, and motor-unit recruitment; similar sEMG amplitudes may therefore produce different torque outputs. Because the chronic group encompassed a wide range of time since injury, this broad classification may have obscured heterogeneity in neuromuscular status among participants with different injury durations. In addition, potentially relevant clinical and behavioral factors, including rehabilitation exposure, preinjury activity level, pain during testing, psychological readiness or fear of reinjury, baseline strength, and hormonal status, were not systematically quantified. These unmeasured factors may have confounded the observed differences in muscle strength and neuromuscular activation. Future studies should incorporate more detailed temporal stratification and prospectively assess these potential confounders. Finally, the study assessed peripheral neuromuscular and biomechanical outcomes without directly measuring central nervous system activity. Multimodal approaches integrating functional near-infrared spectroscopy, sEMG, and kinematic analysis may help link cortical activation to lower-limb muscle coordination (43).

## 5 Conclusion

ACL injury was characterized by lower affected-limb muscle strength and greater antagonist coactivation. Strength deficits were most pronounced in the acute group, whereas coactivation was generally greatest in the chronic group. Increased hamstring coactivation, with greater lateral than medial hamstring coactivation, and altered quadriceps coactivation balance were observed in ACL-injured participants and may be consistent with compensatory neuromuscular patterns related to knee instability. These patterns were associated with lower net force output and worse patient-reported function. Longitudinal and interventional studies are required to determine whether these neuromuscular patterns are modifiable and whether their normalization improves clinical outcomes.

## Data Availability

The original contributions presented in the study are included in the article/Supplementary material, further inquiries can be directed to the corresponding author.
