# Supplementary material for: Functional deficits after anterior cruciate ligament injury are associated with altered neuromuscular control: a cross-sectional study
Source: Front Med (Lausanne). 2026 Sep 16;13:1945491. doi: 10.3389/fmed.2026.1945491 (PMC13623592; doi:10.3389/fmed.2026.1945491)
Supplement: Supplementary file 1 [file Table_1.pdf]

Supplementary Material

Table S1. Limb symmetry indices for maximal voluntary isometric contraction and isokinetic muscle strength.

Outcome	Acute group (n=55)	Chronic group (n=59)	Control group (n=57)	P value
**Extension**				
LSI_{PT} (%)				
Male	56.39±22.31	85.45±18.02	96.12±3.94	<0.001[abc]
Female	42.15±24.87*	72.37±17.93*	92.03±4.08*	<0.001[abc]
LSI_{RTD100} (%)				
Male	45.27±15.36	79.23±17.38	97.64±2.13	<0.001[abc]
Female	34.15±14.71*	65.17±17.92*	94.57±2.52*	<0.001[abc]
LSI_{APT} (%)				
Male	58.79±13.58	78.49±15.29	95.29±2.58	<0.001[abc]
Female	40.69±12.51*	64.26±14.78*	93.17±3.67*	<0.001[abc]
LSI_{AP} (%)				
Male	42.56±10.19	72.51±12.90	95.98±3.25	<0.001[abc]
Female	36.37±9.67*	59.17±13.04*	92.45±2.46*	<0.001[abc]
**Flexion**				
LSI_{PT} (%)				
Male	53.05±10.99	90.21±5.23	90.31±3.21	<0.001[ab]
Female	52.11±11.81	92.15±4.45	91.27±3.32	<0.001[ab]
LSI_{RTD100} (%)				
Male	40.67±9.31	62.63±9.34	91.96±2.15	<0.001[abc]
Female	37.67±10.25	60.15±10.12	91.21±2.05	<0.001[abc]
LSI_{APT} (%)				
Male	61.81±8.53	78.89±10.78	96.27±2.65	<0.001[abc]
Female	51.94±10.35*	69.86±9.54*	93.62±3.78*	<0.001[abc]
LSI_{AP} (%)				
Male	62.59±9.67	63.72±9.27	97.39±2.90	<0.001[bc]
Female	53.61±9.34*	54.89±9.48*	94.67±2.36*	<0.001[bc]

Data are presented as mean ± standard deviation (SD). AP, average power; APT, average peak torque; LSI, limb symmetry index; PT, peak torque; RTD100, rate of torque development from 0 to 100 ms. (a) Significant difference between the acute and chronic groups ($P < 0.05$). (b) Significant difference between the acute and control groups ($P < 0.05$). (c) Significant difference between the chronic and control groups ($P < 0.05$). * Significant difference compared with male participants ($P < 0.05$).

Table S2. Antagonist muscle coactivation outcomes not shown in the main manuscript.

Outcome	Acute group (n=55)		Chronic group (n=59)		Control group (n=57)		P value	
	Affected side	Contralateral side	Affected side	Contralateral side	Non-dominant side	Dominant side	P1	P2
**Extension**								
MH (%)								
Male	8.97±4.51	5.78±4.43	13.38±5.54	6.21±3.95	5.25±3.89	5.01±3.03	<0.001[abc]	0.270
Female	19.59±5.52*	15.77±7.48	26.37±6.15*	18.34±5.81	15.31±4.21*	15.42±4.31	<0.001[abc]	0.493
**Flexion**								
VM (%)								
Male	7.58±3.49	4.19±2.56	7.93±3.02	4.61±1.98	4.59±2.51	4.23±2.31	<0.001[bc]	0.231
Female	10.28±4.37*	7.23±3.52	11.48±3.68*	6.61±3.15	6.92±2.98*	6.88±3.49	<0.001[bc]	0.793

Data are presented as mean ± standard deviation (SD). Group comparisons for affected/non-dominant side and contralateral/dominant side are indicated by P1 and P2, respectively. MH, medial hamstrings. VM, vastus medialis. (a) Significant difference between the acute and chronic groups ($P < 0.05$). (b) Significant difference between the acute and control groups ($P < 0.05$). (c) Significant difference between the chronic and control groups ($P < 0.05$). * Significant difference compared with male participants ($P < 0.05$).

Table S3. Correlations among muscle strength, sEMG parameters, and KOOS subscale scores.

Parameters	KOOS				
	Pain	Symptoms	ADL	Sport/recreation	QoL
MVIC muscle strength					
PT_E	0.523$^{\alpha}$	0.151	0.134	0.434	0.061
$RTD100_E$	0.426$^{\alpha}$	0.331	0.031	0.022	0.127
PT_F	0.329$^{\alpha}$	0.234	0.039	0.419	0.288
$RTD100_F$	0.589$^{\alpha}$	0.229	0.124	0.121	0.184
Isokinetic muscle strength					
APT_E	0.415$^{\alpha}$	0.381$^{\alpha}$	0.485$^{\alpha}$	0.562$^{\alpha}$	0.459$^{\alpha}$
AP_E	0.238	0.351	0.046	0.503$^{\alpha}$	0.149
APT_F	0.229	0.122	0.327$^{\alpha}$	0.325	0.041
AP_F	0.023	0.037	0.129	0.383	0.153
H:Q	0.345	0.425$^{\alpha}$	0.503$^{\alpha}$	0.346$^{\alpha}$	0.218
sEMG					
CH	-0.326$^{\alpha}$	-0.432$^{\alpha}$	-0.487$^{\alpha}$	-0.479$^{\alpha}$	-0.541$^{\alpha}$
LH	-0.591$^{\alpha}$	-0.028	-0.121	-0.026	-0.418$^{\alpha}$
MH	-0.156	-0.055	-0.037	-0.038	-0.029
LH/MH	0.140	0.052	-0.039	0.135	-0.104
CQ	-0.030	-0.244	0.235	-0.212	-0.426$^{\alpha}$
VL	-0.038	-0.045	-0.034	-0.104	-0.120
VM	0.032	-0.137	-0.024	-0.101	-0.314
VL/VM	-0.029	-0.033	0.030	-0.131	-0.201
CH/CQ	-0.150	-0.025	-0.026	-0.383	-0.408$^{\alpha}$

E, extension. F, flexion. ADL, Activities of Daily Living; AP, average power; APT, average peak torque; CH, combined hamstrings; CH/CQ, combined hamstrings to combined quadriceps coactivation ratio; CQ, combined quadriceps; H:Q, hamstring-to-quadriceps ratio; KOOS, Knee injury and Osteoarthritis Outcome Score; LH, lateral hamstrings; LH/MH, lateral hamstrings to medial hamstrings coactivation ratio; MH, medial hamstrings; PT, peak torque; QoL, Quality of Life; RTD100, rate of torque development from 0 to 100 ms; Sport/Rec, Sport and Recreation; VL, vastus lateralis. VL/VM, vastus lateralis to vastus medialis coactivation ratio; VM, vastus medialis. α: Indicates a significant correlation at $P < 0.05$.

Table S4. Correlations between isokinetic muscle strength and sEMG parameters.

Parameters	APT_E	AP_E	APT_F	AP_F	H:Q
CH	-0.505$^{\alpha}$	-0.495$^{\alpha}$	0.081	0.324	0.461$^{\alpha}$
LH	-0.439$^{\alpha}$	-0.431$^{\alpha}$	-0.045	0.115	0.308
MH	-0.251	-0.308$^{\alpha}$	0.052	0.207	0.039
LH/MH	-0.446$^{\alpha}$	-0.398$^{\alpha}$	0.305	-0.041	-0.222
CQ	-0.278	0.182	-0.512$^{\alpha}$	-0.329	-0.465$^{\alpha}$
VL	-0.341$^{\alpha}$	-0.080	-0.318$^{\alpha}$	-0.445$^{\alpha}$	-0.196
VM	0.002	0.159	-0.328	-0.487$^{\alpha}$	-0.141
VL/VM	0.114	0.171	-0.467$^{\alpha}$	-0.115	-0.435$^{\alpha}$
CH/CQ	0.313	-0.270	0.447$^{\alpha}$	0.334$^{\alpha}$	0.492$^{\alpha}$

E, extension. F, flexion. α: Indicates a significant correlation at $P < 0.05$.
